# Necessary Intensity of Monitoring After Elective Craniotomies: A Prediction Score for Postoperative Complications to Stratify Postoperative Monitoring

**DOI:** 10.1007/s12028-025-02242-z

**Published:** 2025-05-22

**Authors:** Elena Kurz, Darius Kalasauskas, Dominik Wesp, Harald Krenzlin, Alicia Schulze, Melek Bulut, Thomas Kerz, Florian Ringel, Naureen Keric

**Affiliations:** 1 Department of Neurosurgery, University Hospital Mainz, Langenbeckstr. 1, 55131 Mainz, Germany; 2 Institute of Medical Biometrics, Epidemiology, and Informatics, University Hospital Mainz, Mainz, Germany

**Keywords:** Postoperative monitoring, Postoperative complications, Elective craniotomy, Prediction, Monitoring capacity

## Abstract

**Background:**

Postoperative complications requiring monitoring following elective craniotomies occur in ~ 2% of cases. Therefore, in most neurosurgical departments, an elective craniotomy is routinely followed by postoperative monitoring in an intensive or intermediate care unit. However, there is no systematic allocation to this procedure. Consequently, patients at risk are not monitored as a priority. The aim of this study was to develop a prediction score for the occurrence of postoperative complications after elective craniotomies and to redefine the monitoring algorithm.

**Methods:**

In this retrospective single-center analysis, all patients with elective craniotomy between 2018 and 2021 were included. Demographic data, diagnosis, location of the pathology (infratentorial/supratentorial), American Society of Anesthesiologists (ASA) score, Charlson comorbidity index (CCI), duration of surgery, blood loss, postoperative complications, and type and duration of monitoring were analyzed. The score was developed and validated internally to ensure its predictive reliability.

**Results:**

A total of 860 consecutive patients (376 male patients and 484 female patients) with a mean age of 60.6 years (range 19–93 years) were included. Forty-three patients experienced a postoperative adverse event that required monitoring. Independent predictors for postoperative complications were age (odds ratio [OR] 0.001, 95% confidence interval [CI] 1.0–1.04), CCI (OR 1.19, 95% CI 1.04–1.36), operative duration (OR 45.90, 95% CI 10.01–229.30), vestibular schwannoma as the treated pathology (OR 1.58, 95% CI 0.09–0.77), blood loss (OR 1.001, 95% CI 1.00–1.001), and ASA score (OR 1.1, 95% CI 1.01–1.2). The score was based on the most reliable characteristics and the calculated predictor error. The formula for score calculation is as follows: 1.3 age + 10 CCI + 65 1_{vascular pathology = yes}_ + 0.5 duration of surgery + 20.5 ASA score − 100. The discriminatory value for clinical outcomes achieved an area under the curve of 0.78 in validation data.

**Conclusions:**

This score provides a practical approach for individual risk assessment of patients undergoing elective craniotomy. Postoperative monitoring capacity can be optimally distributed, and fast-track pathways can be developed for low-risk patients to use this valuable resource effectively.

## Introduction

At most neurosurgical departments, patients undergoing elective craniotomy for intracranial surgery are postoperatively monitored for hemodynamics, respiratory parameters, and neurology on an intensive care unit (ICU) or intermediate care unit (IMC) as a routine practice [1]. Many neurosurgical departments have strictly adhered to postoperative monitoring after intracranial procedures, primarily due to concerns about postoperative intracranial hematoma and edema leading to patients’ decline. These surgical complications typically can occur in the early postoperative period. Nonetheless, literature suggests that only about 2% of electively operated patients require revision surgery or any other intervention for intracranial postoperative hematoma or edema [2–4].

Various neurosurgical patient groups have been evaluated for postoperative complications and the resulting need for intensive care monitoring in the past [4, 5]. Factors such as body mass index (BMI), intraoperative blood loss, and duration of surgery have been identified to have predictive value in some publications [6–8]. Existing scores to predict infections, reoperations, and other adverse events have shown a high predictive value and efficacy in patients with intracranial tumors [9]. Some studies have suggested a lower need for monitoring after elective craniotomy than commonly practiced in most neurosurgical departments, hinting at the applicability of alternative postoperative management strategies [10–13]. However, recommendations for the efficient and reasonable allocation of ICU or IMC beds to neurosurgical patients with elective craniotomies are lacking.

The aim of this study was to evaluate our cohort of patients undergoing elective craniotomy for adverse events and to identify predictors for a high-risk secondary postoperative morbidity that make intensive or intermediate care monitoring necessary and effective. Based on these results, we aimed to develop a guideline for postoperative monitoring after elective craniotomy.

## Methods

The data were acquired through a single-center retrospective analysis. The study included patients who had undergone elective craniotomy for tumors and neurovascular diseases over a 3-year period. The following data were collected and analyzed: demographic information, diagnosis, American Society of Anesthesiologists (ASA) score, Charlson comorbidity index (CCI), duration of surgery, blood loss, complications requiring mandatory monitoring (hemodynamic complications, respiratory failure, decline in neurological status), and the type and duration of monitoring. Univariate and multivariate logistic regression were employed to assess the correlation between potential risk factors and the occurrence of complications. The Benjamini–Hochberg procedure, with a false discovery rate of 5%, was implemented to control for multiple hypothesis testing. The significance level was set to 5%.

For score development and validation, the study data were randomly divided in development (*n* = 602, 70%) and validation (*n* = 258, 30%) data sets. Because of missing values, three patients were excluded from the development group. On the remaining 599 patients, the statistical influence on complications was analyzed using Fisher’s exact test for binary data and the Wilcoxon signed-rank test for continuous data. The most reliable variables (*p* ≤ 0.1) were used for an automatic variable selection procedure based on the Akaike information criterion (AIC) and logistic regression modeling. The linearity assumption was verified for continuous data. As a result, the ASA score was squared, and hours were logarithmically included in the starting model. The regression coefficients of the selected model were converted into an easy-to-use scoring system. We followed the *Transparent Reporting of a multivariable prediction model for Individual Prognosis Or Diagnosis* (TRIPOD) guidelines [14] for the development, validation, and reporting of the proposed score. The final model’s discrimination was assessed in both the development and validation data using areas under the receiver operating characteristic curve (AUROCs). Calibration was assessed on the development data. Additionally, the score’s performance was evaluated on both data sets including AUROCs. The score was finally used to create a classifier distinguishing between patients at low and high risk of postoperative complications. The Jaccard index and the Youden index led to a sensitivity < 0.7. Therefore, the score was chosen by maximizing the specificity, keeping the minimal sensitivity to 0.8.

Based on the internal validation results, a three-tiered risk classification was devised, with low-risk (0–4% risk for a postoperative complication, 0–113 points), intermediate-risk (5–15%, 104–150 points) and high-risk (> 15%, > 150 points) levels corresponding to the respective range of points. These cutoffs were chosen based on clinical risk evaluation. This study was approved by the local review board.

## Results

### Patients’ Characteristics

A total of 860 consecutive patients were included in the study, comprising 376 men (43.7%) and 484 women (56.3%). All patients were monitored postoperatively on our ICU or IMC. The mean age was 60.6 years (range 19–93 years). In the preoperative anesthesiologic premedication, patients were assigned a mean ASA score of 2.5. The distribution of grades was as follows: grade 1, *n* = 10 (1.2%); grade 2, *n* = 423 (49.2%); grade 3, *n* = 380 (44.2%); and grade 4, *n* = 47 (5.4%). The median CCI for the patient cohort was 2 points (1 point, *n* = 50; 2 points, *n* = 400; 3 points, *n* = 61; 4 points, *n* = 60; 5 points, *n* = 14; 6 points, *n* = 180; 7 points, *n* = 35; 8 points, *n* = 35; 9 points, *n* = 17; 10 points, *n* = 5; 11 points, *n* = 2; 16 points, *n* = 1).

The majority of patients underwent elective microsurgical resection of intra-axial tumors (*n* = 515, 59.9%). In 93 cases, the surgical procedure was performed because of a vascular pathology. The remaining patients underwent craniotomy as part of the following pathologies or procedures: cranioplasties, pituitary adenomas, and skull base reconstructions. These causes for craniotomy are summarized under others in Table 1. A total of 276 (32.1%) patients were treated for an intrinsic tumor. In 708 cases (82.3%), the pathology was located supratentorially, whereas in 152 cases (17.7%), it was located infratentorially. Further detailed information on the patients’ characteristics is provided in Table 1.

**Table 1:** Patients’ characteristics and preoperative scores and diagnoses that led to elective craniotomy

Characteristics	Patients
Number	860
Sex, *n* (%)	
Male	376 (43.7)
Female	484 (56.3)
Age, mean (SD), yr	60.6 (14.5)
ASA score, median points	2
CCI, median points	2

Pathology, *n* (%)	Patients
Intrinsic tumors	276 (32.1)
Metastases	231 (26.9)
Meningioma	193 (22.4)
Vascular pathologies	93 (10.8)
Schwannoma	23 (2.7)
Others^a^	44 (5.1)

*ASA* American Society of Anesthesiologists, *CCI* Charlson comorbidity index

^a^Hemangioblastoma, colloidal cyst, liponeurocytoma, craniopharyngioma, cavernoma, ependymoma, arachnoid cyst, lymphoma, hemangiopericytoma, cerebellar cyst, plexus papilloma, cholesteatoma, epidermoid, pineal cyst, and hemangioma

### Surgery and Postoperative Monitoring

The mean duration of surgery was 191 min (SD 89.9 min) for all procedures in general, whereas the mean blood loss was 298 mL (SD 411.5 mL). All patients were monitored on our ICU (*n* = 545) or IMC (*n* = 315). The mean monitoring time, excluding patients with postoperative complications, was 26.6 h (SD 35.3 h). However, the mean monitoring time for patients with complications was 67.3 h (SD 146.5 h).

It was standard practice to transfer patients to the ward between 10 a.m. and 12 a.m. in the morning. Eighty-eight patients were transferred to the ward during the night with the purpose to make an ICU bed available for the admission of emergency cases. There was no significant correlation between the time of transfer and the development of a complication (*p* = 0.46).

### Postoperative Complications

A total of 119 (13.8%) patients experienced a postoperative incident that necessitated at least IMC monitoring. In the majority of cases, patients exhibited impaired or rapidly deteriorating alertness. In these instances, an emergent computed tomography (CT) scan was performed to rule out postoperative hemorrhage or edema causing increased intracranial pressure. In 24 (2.8%) patients, a postoperative hemorrhage was identified. Eight of these patients had additional relevant edema, whereas two patients demonstrated the initial signs of hydrocephalus on the CT scan. Twenty (2.3%) patients underwent revision surgery. Respiratory failures and pulmonary complications were the reason for a readmission on the ICU in 77 (8.9%) patients. Respiratory failure was caused by several factors, including pulmonary embolism (*n* = 4), pneumonia (*n* = 41), aspiration due to dysphagia (*n* = 5), a reduced respiratory drive resulting from an impaired neurological status (*n* = 27), hydropic decompensation (peripheral and pulmonary edema, pleural effusion) (*n* = 12), and others. These factors are collectively referred to as “respiratory complications.” Seizures and surgical complications (hemorrhage, edema, hydrocephalus) were responsible for an impairment of alertness that required monitoring in 42 cases (Table 2).

**Table 2:** Postoperative complications in the first 24 h that led to extended monitoring

Complications	Cases
All, *n* (%)	119 (13.8)
Impaired alertness/neurological deficit	
Hemorrhage, *n* (%)	24 (2.8)
Edema, *n* (%)	8 (0.9)
Infarction, *n* (%)	8 (0.9)
Hydrocephalus, *n* (%)	2 (0.2)
Seizures, *n* (%)	10 (1.2)
Revision surgery, **n** (%)^a^	22 (2.3)
Respiratory failure (NIV/intubation)	77 (8.9)

*NIV* noninvasive ventilation

^a^Twenty revision surgeries resulted from surgical complications

**Fig. 1:**
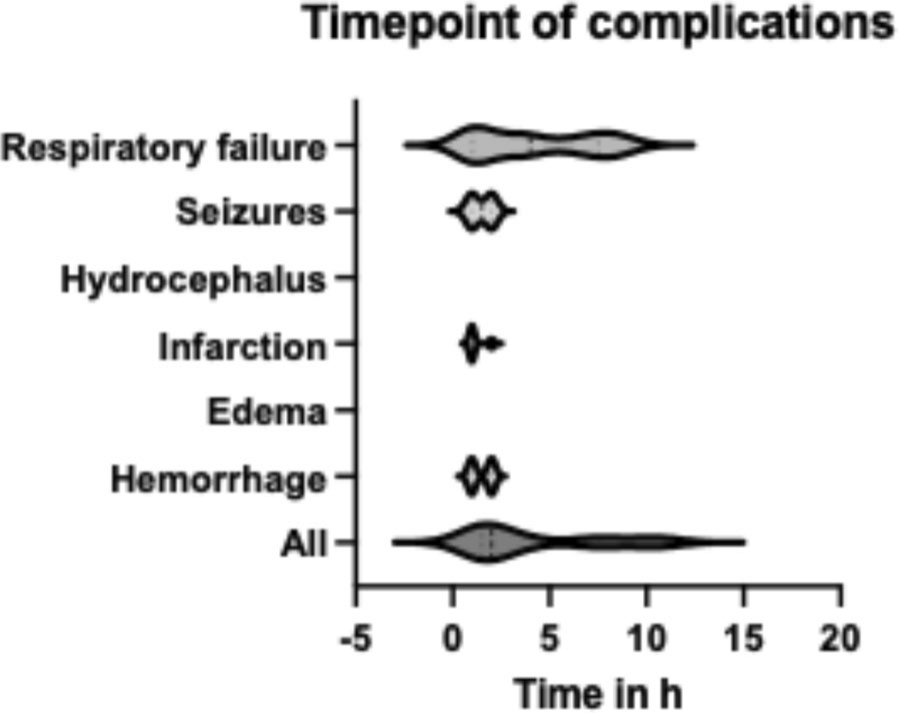
Violin plot showing time point of complications. The mean time point of complications was 5.0 h postoperatively. SD 3.6 h

Ten patients died during their hospital stay as a consequence of their initial disease (*n* = 3), decompensation of previous illnesses (*n* = 3), or cardiac complications (*n* = 4). In 50% of all patients with a complication that warranted monitoring, the adverse event occurred 5.0 h (SD 3.5 h) after transfer to the ICU/IMC postoperatively. To capture 90% of complications, monitoring in the ICU or IMC for 12 h would be required (Fig. 1). Patients who developed postoperative hemorrhage were identified on average 7.7 h (SD 6.5 h) after surgery, whereas pulmonary complications occurred on average 5.8 h (SD 4.5 h) after surgery. A total of 242 patients were monitored more than 24 h on the ICU or IMC (mean 70.9 [SD 119.0] hours), whereas 612 patients were monitored for less than 24 h (mean 18.1 [SD 4.6] hours). There was no evidence that patients monitored less than 24 h had a higher risk of postoperative complications.

Fourteen patients were readmitted to the ICU because of revision surgery after a mean time of 130.8 h (SD = 143.4).

### Risk Factors

Univariate analysis based on the whole patient group revealed that sex (*p* = 0.99), certain types of pathology (such as intrinsic tumor [*p* = 0.54], metastases [*p* = 0.66], or meningioma [*p* = 0.79]), the location of the pathology (*p* = 0.62), BMI (*p* = 0.75), and the level of monitoring (ICU, IMC, or postanesthetic care unit) (*p* = 0.67) did not influence the risk for postoperative complications. No correlation was found between the time of transfer to the ward and the development of complications (*p* = 0.46).

Significant associated factors in univariate analysis are listed in Table 3. The following factors correlated significantly with the development of postoperative complications: age, the ASA score, the CCI, presence of an *Obstructive Sleep Apnea Syndrome* (OSAS), whether the treated pathology was a vascular kind or a schwannoma, the duration of surgery, blood loss, and the duration of postoperative monitoring on the ICU/IMC.

**Table 3:** Univariate logistic regression analysis significantly correlating parameters with the development of postoperative complications, increasing risk per unit or grade

Variable	*p* value	OR	95% CI
Age	0.001	1.025	1.01–1.04
ASA score	< 0.0001	1.95	1.42–2.67
CCI	0.006	1.112	1.03–1.21
Vascular pathology	0.05	1.58	1.34–1.58
Duration of surgery (per 10 min)	< 0.0001	1.01	1.01–1.01
Blood loss (per 100 mL)	< 0.0001	1.074	1.03–1.116
OSAS	0.04	0.28	0.08–0.97
Schwannoma	0.005	0.29	0.11–0.69
Time in ICU or IMC (per h)	0.036	1.004	1.00–1.007

*ASA* American Society of Anesthesiologists, *CCI* Charlson comorbidity index, *CI* confidence interval, *ICU* intensive care unit, *IMC* intermediate care unit, *OR* odds ratio, *OSAS* obstructive sleep apnea syndrome

In the multivariate analysis based on the whole cohort, six parameters with an impact on the risk for postoperative complications were identified: CCI (*p* = 0.006, odds ratio [OR] 1.21, 95% confidence interval [CI] 1.06–1.38), ASA (*p* < 0.0001, OR 1.5, 95% CI 0.94–2.32), age (for every year) (*p* = 0.001, OR 1.02, 95% CI 1.004–1.045), duration of surgery (per 10 min) (*p* < 0.0001, OR 1.009, 95% CI 1.007–1.012), schwannoma as the treated pathology (*p* = 0.015, OR 1.58, 95% CI 0.09–0.77), and blood loss (per 100 mL) (*p* ≤ 0.0001, OR 1.001, 95% CI 1.00–1.001). An overview is shown in Table 4.

**Table 4:** Multivariate logistic regression analysis significantly correlating parameters with the development of postoperative complications, increasing risk per unit or grade

Variable	*p* value	OR	95% CI
Age	0.001	1.025	1.01–1.04
ASA score	< 0.0001	1.95	1.42–2.67
CCI	0.006	1.112	1.03–1.21
Schwannoma	0.015	1.58	0.09–0.77
Duration of surgery (per 10 min)	< 0.0001	0.3	1.01–1.01
Blood loss (per 100 mL)	< 0.0001	1.001	1.00–1.001

*ASA* American Society of Anesthesiologists, *CCI* Charlson comorbidity index, *CI* confidence interval, *OR* odds ratio

The threshold for an increased risk of complications was duration of surgery of 195 min (Youden score 0.32). Patients who experienced intraoperative blood loss of greater than 550 mL were more likely to develop postoperative complications (Youden score 0.15). Furthermore, the CCI threshold for higher complication rates was greater than 6 points (Youden score 0.11).

The risk increased with each additional ASA score grade, with an additional 50.5% risk per grade to develop a postoperative complication. Every CCI grade was found to have an impact of 21% on the risk of developing a complication, whereas a 10-year age difference caused a risk growth of 20%. Furthermore, an additional 8% risk can be attributed to a 10-min increase in the time from skin incision to closure. Similarly, a 10% increase in the risk can be attributed to every 100 mL of blood loss.

### Prediction Score

Based on the evaluated parameters, we aimed to create a prediction score to identify patients with a high risk for postoperative complications. The score was developed and validated internally. A total of 599 patients were included in the development group, whereas 258 patients were used to validate the score.

**Fig. 2:**
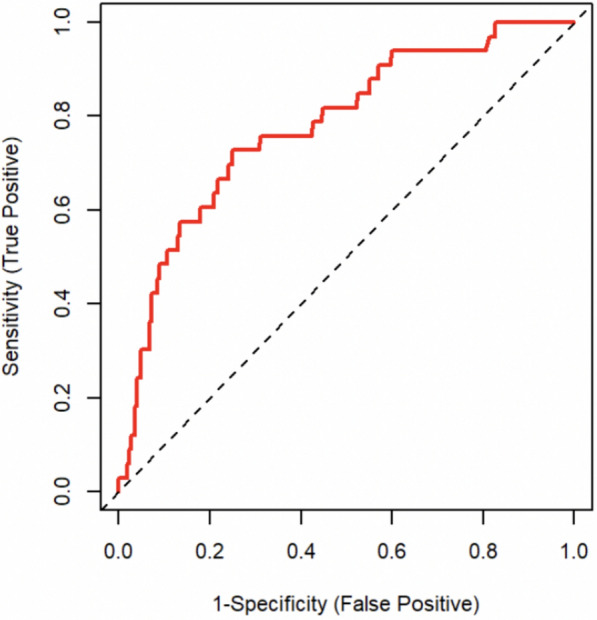
Receiver operating characteristic analysis of internal validation of the developed prediction score. Area under the curve = 0.78

A score was created based on the AIC and the Bayesian information criterion, with the most reliable variables (*p* ≤ 0.1) selected to form the score. The parameters used for the development of the score (with a *p* ≤ 0.1) were the age, the CCI, the ASA score, infratentorial/supratentorial location, vascular pathology, schwannoma, duration of surgery, and blood loss. Multivariate logistic regression analysis using AIC identified age (OR 1.024, 97.5% CI 1.004–1.045), the CCI (OR 1.21, 97.5% CI 1.06–1.38), the ASA score (OR 1.47, 97.5% CI 0.94–2.32), the duration of surgery (OR 1.009, 97.5% CI 1.007–1.012), and the presence of vascular pathology (OR 3.42, 97.5% CI 1.54–7.4) as the best set of predictors of postoperative complication. Based on these parameters, the score showed an AUROC of 0.78 in the analysis of the validation data (Fig. 2).

The final model’s performance was evaluated on both the development and validation data sets using a range of measures, including the AUROC for discrimination and the calibration line intercept and slope for calibration. To address the potential issue of overfitting, we conducted internal validation with 1,000 bootstrap samples. Additionally, we assessed the discriminative performance of the scoring system derived from the final model on both the development and validation data sets.

A score of 137.9 points was calculated as a cutoff, indicating a higher risk of postoperative complications for higher values independently of the corresponding score value. The sensitivity of the cutoff was 0.8/0.79, the specificity was 0.53/0.56, the positive predictive value was 0.22/0.21, and the negative predictive value was 0.94/0.95 in the development/validation data, respectively. The formula for calculating the scoring system is as follows: 1.3 age + 10 CCI + 65 1_{vascular pathology = yes}_ + 0.5 duration of surgery + 20.5 ASA score − 100. The sensitivity and specificity for the two cutoffs were 0.94 and 0.24 for the cutoff at 5% and 0.67 and 0.75 for the cutoff of 15%, respectively (Table 5).

**Table 5:** Prediction score for postoperative complications after elective craniotomy

Age (years)	Points	Duration of surgery (min)	Points	Vascular surgery	Points
18–29	26	30–89	15	Yes	65
30–39	39	90–149	45	No	0
40–49	52	150–209	75	**CCI**	**Points**
50–59	65	210–269	105	Per point	10 (max 240)
60–69	78	270–329	135		
70–79	91	330–389	165		
80–89	104	390–449	195		
> 90	117	450–509	225		
**ASA**	**Points**	510–569	255	**Grading** (risk of complication)	
1	20.5	570–629	285	Maximum score	879
2	41	630–689	315	Low risk (0–4%)	0–103
3	61.5	690–749	345	Intermediate risk (5–15%)	104–150
4	82.5	> 750	375	High risk (> 15%)	> 150

Included parameters: CCI, ASA score, age, duration of surgery, and vascular pathology. Risk evaluation was divided into three groups: low risk (0–4%), intermediate risk (5–15%), and high risk (> 15%)

*ASA* American Society of Anesthesiologists, *CCI* Charlson comorbidity index, max, maximum

## Discussion

Monitoring capacities have always been a very limited commodity. Efficient risk-based distribution to patients is not always easy in day-to-day clinical practice. In most neurosurgical departments patients are monitored on the ICU or IMC after elective craniotomies. However, there are no recommendations for the duration and kind of postoperative monitoring for patients concerned. Consequently, patients at risk are not monitored as a priority.

In this study, we were able to develop a prediction score that provides a practical approach for individual risk assessment of patients undergoing elective craniotomy. This score can improve the efficiency of the allocation of the valuable resource of monitoring capacities by prioritization of patients at high risk for postoperative complications after elective craniotomies.

In our cohort, in 13.8% of cases, a complication requiring monitoring occurred. The majority (64.7%) of the complications had a pulmonary cause. We found that a 12-h monitoring period is sufficient to capture 90% of all incidents. We identified age, the CCI score, duration of surgery, vestibular schwannomas as the treated pathology, blood loss, and the ASA score as independent predictors of postoperative complications. However, the combination these predictors, excluding blood loss, formed the best prediction model for postoperative complications with regard to the AIC. Based on these parameters, we developed the presented three-tiered risk classification and validated it internally. This score can then be employed for a preoperative risk stratification, thereby optimizing the use of ICU beds and prioritizing patients with a calculated high risk.

The postoperative monitoring of patients who have undergone elective neurosurgical intracranial procedures is a routine standardized procedure that is conducted in most neurosurgical departments. It is standard practice for hospitals to have established standard operating procedures, which provide guidance on bed allocation and the duration of monitoring. However, these workflows are not standardized and vary in international and national comparison. The decision-making process is based on internal agreements and the experiences of senior neurosurgeons and anesthetists.

Nevertheless, some attempts have been made to evaluate the necessity of ICU monitoring for neurosurgical patients, while clear recommendations have not been established so far [4, 5, 9, 15]. Sioshansi et al. analyzed their cohort of 200 patients after lateral skull base approaches and found that 17 (8.5%) of them experienced an adverse event. This was in the context of mandatory intensive care monitoring in patients. It is notable that the majority of complications were described as hypertensive urgencies. There were no mortalities [5].

Ziai et al. analyzed a group of 158 patients after cranial tumor resection and found that of 158 patients, 23 required monitoring in the ICU for more than 24 h. As potential predictors for the necessity to exceed 24-h monitoring due to complications or instability, radiologic findings, large intraoperative blood loss, fluid requirements, and the decision to keep the patient intubated at the end of surgery were identified [4].

A group of patients undergoing elective craniotomies was evaluated by Bui et al. They found that 12.5% of 343 patients were planned for postoperative ICU monitoring because of the length of surgery or anesthetic risks. In only 2% of cases, an unexpected admission or transfer to the ICU was necessary because of slow neurological recovery and extensive intraoperative blood loss. In 3% of cases, a medical emergency team was called for the management of complications on the ward [15]. In the study by Lonjaret et al. 16% of patients presented with a neurological complication, which may justify monitoring for early detection [16]. In a review of literature from 2001 to 2021, testing alternative postoperative pathways excluding ICU monitoring, Azad et al. found that only 2% of 2469 patients needed a readmission to the ICU [17].

The present analysis focused on complications that occurred within the first 24 h following craniotomy. The rationale for selecting this temporal range is that the early postoperative phase is commonly perceived as a period of heightened risk for postoperative hematoma, edema, and the eventual need for hemodynamic support [18]. As shown in the Results, the mean monitoring time at our department was 26.6 h. Although there is no recommendation for the duration of stay on the ICU/IMC, our monitoring times appear to align with the commonly held understanding of the early postoperative phase as a period of increased risk [3].

In our study the complication rate was 13.8%. A majority (64.7%) of complications were pulmonary in nature, such as pneumonia (34.5%) and hydropic decompensation (peripheral and pulmonary edema, pleural effusion) (10.1%). A minor proportion (2.8%) required monitoring because of neurological deterioration resulting from edema, minor hemorrhage, or hydrocephalus. Twenty patients underwent revision surgery owing to hemorrhage, edema, hypoperfusion, and hydrocephalus whereas ten were readmitted because of generalized seizures causing impaired consciousness and respiratory failure.

### Risk Factors/Prediction Factors

It is already known that certain factors increase the risk of postoperative complications. Sioshansi et al. conducted an analysis of a cohort of patients with purely oncological diagnoses undergoing neurosurgical procedures. Their findings indicated that tumor mass and preexisting hypertension were independent predictors of adverse outcomes [5]. Lohmann et al. identified the number of secondary diagnoses, BMI, and incision closure time as risk factors for adverse events in their evaluation of elective craniotomies [9]. Lonjaret et al. observed that patients undergoing posterior fossa surgery were more likely to be readmitted to the ICU and proposed that ICU monitoring should be prolonged following this type of surgery [16]. Our data do not support this statement, as the location of supratentorial or infratentorial showed only a trend of correlation with the development of complications.

In our study, six factors demonstrated predictive value in the multivariate analysis: CCI, ASA score, the patients’ age, schwannoma as the treated pathology, duration of surgery, and blood loss. The first four parameters were associated with the patients’ conditions and were known preoperatively. These factors could permit the preliminary allocation of monitoring capacities to be made based on preoperative data. The latter two parameters can be evaluated during the operative procedure or subsequently. A prolonged surgical procedure and significant blood loss would justify postoperative monitoring, even in the absence of high-risk stratification, by using solely preoperative factors.

It is noteworthy that other parameters, such as the type of surgery, the location of the intracranial pathology, BMI, obstructive sleep apnea, sex, and the level of postoperative monitoring (IMC or ICU) did not correlate with the risk for postoperative complications. Interestingly, the risk for a complication increased with the time of monitoring. This could depict the correct choice based on experience and clinical aspects to monitor certain patients postoperatively and not to transfer them too early to the ward.

### Prediction Score

Badenes et al. aimed to define criteria to allocate ICU beds to neurosurgical patients at risk of complications [19]. Moreover, an attempt was made to evaluate the parameters with the greatest impact. Nevertheless, the evidence available was insufficient to create a standardized protocol. Lohmann et al. created a *Surgical Outcome Risk Score* (SOS) score to predict postoperative complications in neurooncological patients with intracranial tumors. The score included the size of the tumor, BMI, and the time required from incision to closure. The authors subsequently evaluated an existing score systems on a group of patients with spinal or cranial tumors with a view to predict infections, the risk of reoperation, and adverse events. The *Nursing care level, length of stay on the ICUscore* (NonInfECT), *Leukocytosis, ECOG on admission, Urgency of surgery and Cutting-suture time of index surgery* (LEUCut), and *Leukocytosis, length of stay in the ICU, Nursing care level, and CRP on admission* (LINC) score systems were found to be effective in predicting the occurrence of complications of various types [9]. An external validation of the score for assessment of predictive value is pending [9].

In the present study, it was possible to calculate a threshold for three of the identified parameters that leads to a significantly higher risk for complications. The calculated threshold of approximately 200 min from incision to closure can be used to stratify patients into a high-risk group preoperatively if the planned duration of the operation exceeds this value. In addition, it can be employed for intraoperative decision-making in the event of unexpected prolongation of the operative duration. In the event of blood loss exceeding approximately 500 mL, admission to the ICU should be considered. Furthermore, a CCI of more than 6 points was identified as the level of comorbidities that is associated with a higher perioperative risk and could be used preoperatively as a cutoff for decision-making.

Considering these new insights, it was imperative to conduct a validation and weighting of influencing factors to create a reliable prediction score. In the course of an extended statistical analysis, the prediction score was the most reliable when based on age, CCI, duration of surgery, ASA score, and treatment of a vascular pathology. The model demonstrated a satisfactory predictive capacity, with an AUROC of 0.78. The scoring system allows the determination of the grade of risk for postoperative complications. The score encompasses parameters delineating the patients’ health status, the nature of the pathology, and the duration of surgery, which represents an element of intraoperative influence. It was our objective to provide a reliable assessment of the risk of developing postoperative complications. The score is easy to calculate, and the requisite parameters are used in clinical practice.

### Time of Intensive Care Treatment/Observation

Regarding the results, the period with the highest risk for adverse events was the first postoperative 5.7 h. An SD of 4.4 h indicates that a maximum of 14.5 h of monitoring was necessary to detect 97.5% of complications. The mean monitoring time in our cohort was approximately 26 h, which is more than 100% longer than the expected duration.

In their analysis, Mirza et al. examined 421 patients, of whom 25 had an indication for ICU admission and were already planned for this. Only four patients who were transferred postoperatively to the general ward had to be readmitted to the ICU because of complications. All these complications occurred during the first postoperative hours. The study concluded that after 4 h of monitoring in the postanesthetic care unit, the risk of a severe complication was very low [11].

A similar study was conducted by Pendharkar et al., which involved the transfer of 63 young patients (aged < 65 years and ASA class of 1, 2, or 3) after elective craniotomy to the ward for hourly neurological examination for 8 h. Only 11% of patients were admitted to the ICU. No complications were observed after 8 h [13].

In light of the findings of other published studies and the present investigation, it can be concluded that a monitoring period exceeding 10 h is not obligatory. However, to detect 90% of all incidents, a monitoring time of 12 h is warranted. To capture 90% of the complications, the mean time of 5.5 h needs to be added to twice the SD (5.5 h + 2 × 3.5 h). The implementation of a guideline regulating the time of monitoring and a subsequent rapid transfer to the ward would have a considerable impact on the valuable resources of ICU or IMC capacity and specialized medical personnel.

## Limitations

There are some limitations to this study. Firstly, the retrospective character of the study can cause a selection bias, and our study is a single-center evaluation. The generalizability of the results is limited because the results are based on a cohort of patients treated in the same environment and with the same treatment protocol. Additionally, the patient group was rather heterogeneous because all electively treated pathologies were included. Concerning the prediction score, we conducted an internal validation using separate discover and validation data sets. However, no external validation was made with an independent patients’ collective.

## Conclusions

This study shows that postoperative ICU monitoring beyond 12 h is not necessary in most cases, as most complications occur during this early postoperative period. To identify high-risk patients, it is necessary to consider comorbidities, age, and other surgery-related factors. Taking these significant factors into account, a risk assessment score was developed and internally validated for this patient group. An external validation of the presented prediction score is necessary to confirm the value of the score. It may then be employed prospectively to perform a preoperative risk stratification, thereby optimizing the use of ICU beds and enabling a fast-track recovery pathway for patients.

## Data Availability

All data sets analyzed in this study are available upon reasonable request from the corresponding author.
